# Laser Photobiomodulation 808 nm: Effects on Gene Expression in Inflammatory and Osteogenic Biomarkers in Human Dental Pulp Stem Cells

**DOI:** 10.3389/fphar.2021.782095

**Published:** 2022-01-17

**Authors:** Elaine A. da Rocha, Marcela M. P. Alvarez, Agatha M. Pelosine, Marcela Rocha O. Carrilho, Ivarne L. S. Tersariol, Fábio D. Nascimento

**Affiliations:** ^1^ Technology Research Center, Mogi das Cruzes University, Mogi das Cruzes, Brazil; ^2^ Department of Biochemistry, Federal University of São Paulo, São Paulo, Brazil; ^3^ Interdisciplinary Center of Biochemical Investigation, University of Mogi das Cruzes, Mogi das Cruzes, Brazil; ^4^ College of Dental Medicine-Illinois, Midwestern University, Downers Grove, IL, United States

**Keywords:** human dental pulp stem cells, photobiomodualtion therapy, gene expreesion, dental pulp stem cells, inflammation, bone osteogenesis

## Abstract

The tissue engineering of dental oral tissue is tackling significant advances and the use of stem cells promises to boost the therapeutical approaches of regenerative dentistry. Despite advances in this field, the literature is still scarce regarding the modulatory effect of laser photobiomodulation (PBM) on genes related to inflammation and osteogenesis in Postnatal Human Dental Pulp Stem cells (DPSCs). This study pointedly investigated the effect of PBM treatment in proliferation, growth and differentiation factors, mineralization, and extracellular matrix remodeling genes in DPSCs. Freshly extracted human third molars were used as a source for DPSCs isolation. The isolated DPSCs were stimulated to an inflammatory state, using a lipopolysaccharide (LPS) model, and then subjected or not to laser PBM. Each experiment was statistically evaluated according to the sample distribution. A total of 85 genes related to inflammation and osteogenesis were evaluated regarding their expression by RT-PCR. Laser PBM therapy has shown to modulate several genes expression in DPSCs. PBM suppressed the expression of inflammatory gene TNF and RANKL and downregulated the gene expression for VDR and proteolytic enzymes cathepsin K, MMP-8 and MMP-9. Modulation of gene expression for proteinase-activated receptors (PARs) following PBM varied among different PARs. As expected, PBM blocked the odontoblastic differentiation of DPSCs when subjected to LPS model. Conversely, PBM has preserved the odontogenic potential of DPSCs by increasing the expression of TWIST-1/RUNEX-2/ALP signaling axis. PBM therapy notably played a role in the DPSCs genes expression that mediate inflammation process and tissue mineralization. The present data opens a new perspective for PBM therapy in mineralized dental tissue physiology.

## Introduction

A primary objective of regenerative medicine and tissue engineering is to support the reinstatement of tissues and/or organ’s functions by means an *in situ* substitution/repair of their injured structures. However, intrinsic morphological complexities such as those found, for instance, in dental pulp can substantially compromise this tissue successful remodeling and repair. Dental pulp is a unique and specialized mesenchymal tissue that, by being confined inside a mineralized rigid chamber in the core of teeth, has limited or virtually no chance to expand as part of the inflammatory response to accommodate an uneventful tissue turnover. Stromal fibroblasts and odontoblasts are the main regenerative/formative cells in dental pulp tissue (Nor, 2006). Interestingly, the pulp tissue also harbors mesenchymal stem cells with self-renewal capacity and multidifferentiation potential (Botelho et al., 2017).

Postnatal stem cells have proven to be an excellent resource in regenerative medicine. Among mesenchymal postnatal cells found in dental pulp, the Dental pulp stem cells (DPSCs) (Kerkis et al., 2006) have shown to exhibit promising tissue regenerative cues, such as a more mature phenotype in comparison to stem cells derived from exfoliated deciduous teeth (SHEDs) (Goldberg and Smith, 2004), and higher plasticity (i.e. proliferative and differentiation capacity to turn into various cells) than Bone marrow stem cells (BMSCs) (Miura et al., 2003).

Overall, the inflammatory process is generally accompanied by imbalance in gene expression and signaling, as well as by the release of proinflammatory cytokines, such as tumor necrosis factor-alpha (TNF-α), reactive oxygen, nitrogen species and, interleukin-1β (IL-1β) and IL-6 (Hanisch, 2002). LPS can activate the NF-κB signalling pathway in DPSCs. NF-κB is a transcription factor that regulates a large variety of inflammatory cytokines, including TNF-α, IL-1, IL-6, and IL-8 (Baldwin, 2001). The LPS induces the production of pro-inflammatory cytokines in dental pulp fibroblasts (Nakane et al., 1995). Also, it has been shown that LPS can promote odontoblastic differentiation of human DPSCs via TLR-4, ERK, and P38 MAPK signalling pathways (He et al., 2015). The inflammatory interleukin(s)-1. -6. -11 (IL-1. IL-6. IL-11), and TNF-α can stimulate osteoclast development and thereby the process of bone resorption (Manolagas, 1995).

In addition to these canonical inflammatory pathways, cell signaling *via* Proteinase-activated receptors (PARs) are emerging as an important path in the study of inflammatory responses in various tissues (Russell and McDougall, 2009). PARs are part of the family of G-protein-coupled receptors that are activated by proteinases secreted into extracellular matrix (ECM) during inflammation. Whilst first described as thrombin receptors, various other proteinases are able to signal *via* PARs. While PAR-1, PAR-3, and PAR-4 are canonically activated by thrombin, PAR-2 is mainly activated by trypsin. Furthermore, it can be activated by tryptase, matrix metalloproteinases (MMPs), and tissue factor-VIIa-Xa complex (Vu et al., 1991; Bohm et al., 1996; Ruf and Mueller, 2006). PARs are also known to participate on extracellular matrix pathophysiological processes. Collagenase hydrolysis showed an antagonistic behavior on PAR2 activation, proposing an relevant negative feedback mechanism whereby canonical PAR2 activation induces MMP expression, and MMP activity can subsequently antagonize PAR2 (Falconer et al., 2019). PARs 1 and 2 have been recently demonstrated to play a role in inflamed odontoblasts (Alvarez et al., 2017).

Low-level laser therapy, also known as Photobiomodulation Therapy (PBM) (Hamblin, 2016), had its onset in the 1960’s and it relies on the use of light devices–lasers or light-emitting diodes (LEDs) - as resource to trigger tissue biological/medical responses (i.e. healing, immunity enhancement, anti-inflammatory and antibiotic properties) (Vu et al., 1991; Conlan et al., 1996; Bjordal et al., 2003; Yu et al., 2003; Ruf and Mueller, 2006; Ghanaat, 2010; Alvarez et al., 2017; Falconer et al., 2019). Studies have showed that PBM can significantly reduce inflammation, by inhibiting inflammatory cytokines expression and activity in different tissues (Sakurai et al., 2000; Arany et al., 2007; de Lima et al., 2011). Moreover, the use of lasers and LEDs demonstrated to be effective in modulating the cell viability and growth in different cell models, including mesenchymal stem cells (Kim et al., 2009; Li et al., 2010; Alghamdi et al., 2012; Ginani et al., 2015). Research that evaluated the effect of PBM on SHED cells has shown for instance that infrared LEDs (850 nm 40mW/cm^2^) could promote an *in vitro* increase in the levels of phosphates, synthesis of collagen and dentinal sialoprotein (Turrioni et al., 2014), and induce a significant increase in cells viability, proliferation, and production of mineralized tissues for SHEDs that remained in nutrient starvation after PBM (Turrioni et al., 2015).

To better understand DPSC’s features for tissue regenerative applications, such as vital pulp therapy or regenerative endodontic procedures, we believe it is important to assess their biological responses when exposed to light sources under PBM parameters. As far as we know, this is the first study that assessed the potential for laser irradiation to modulate the gene expression for PARs and other genes related to the inflammatory process and osteogenesis using a DPSCs model. This study hypothesis was that PBM can interfere in inflammatory gene expression and in osteogenesis related genes.

## Methods

### Dental Pulp Tissues Obtainment

Approval for this protocol was obtained from the local Human Research Ethics Committee (# 98511618.8.0000.5497) to use five freshly extracted third molars from patients ages 19–39 years. The use of the third molars is the most convenient source of adult stem cells as they contain sufficient amount of dental pulp tissue to ensure proper isolation of DPSCs (Liu et al., 2006). After extraction teeth were copiously washed with deionized water and placed in a sterile solution containing saline, subsequently they were rinsed with 70% ethanol to reduce the biofilm contamination. Then, the dental elements were rinsed 5 times with sodium phosphate buffer (PBS) to remove ethanol. Subsequently, decontaminated teeth were cut with a sterile Zekrya (Dentsplay Sirona, United States) drill using a high-speed device to expose the pulp chamber and, consequently, provide access to the pulp tissue. The pulp tissue was gently removed from the pulp chamber with a sterile endodontic file and immediately transferred to a sterile screw-capped tube containing α-MEM cell culture medium without calf bovine serum (FCS).

### Isolation of Postnatal Human Dental Pulp Stem Cells

Excised pulp tissue was digested in a solution containing 3 mg/ml collagenase type I (Merck KGaA, Darmstadt, Germany) and 4 mg/ml dispase (Merck KGaA, Darmstadt, Germany) for 1 h at 37°C. After digestion, 5 volumes of α-MEM medium containing 10% FCS were added. This solution was centrifuged at 120 × g for 10 min, at room temperature. The precipitated material was resuspended in α-MEM medium and filtered through filters with pores of 70 μM. This procedure resulted in a single-cell suspension for *in vitro* culture. The cells were seeded into culture flasks with α-MEM, supplemented with 10% FCS, 100 μM l-ascorbic acid, 2 mM l-glutamine and penicillin (100 U/mL)/streptomycin (100 mg/ml) and incubated at 37°C with 5% CO_2_. In the initial stage, after adhering to culture flasks, cells grew slowly. Cell colonies were identified after 10–14 days, with their fibroblastoid appearance, which showed to be dependent on the cell density obtained in initial plating. After reach 70% of confluence, cells were considered ready to proceed with experiments. Accordingly, isolated DPSCs were randomly assigned to the following experimental groups: Group 1–LPS stimulus and laser irradiation; Group 2–LPS stimulus and no laser irradiation; Group 3–Control group, no LPS or laser treatment.

### Human Dental Pulp Stem Cells Inflammation Induction by Lipopolysaccharides Assay

The lipopolysaccharides induces the production of pro-inflammatory cytokines such as interleukins (IL-1β, IL-6 and IL-8), tumor necrosis factor (TNF-), platelet and prostaglandin activating factors by macrophages and neutrophils present in areas infected by bacteria (Lindemann et al., 1988; Wilson et al., 1996). To promote *in vitro* cellular inflammatory response, 2 × 10^6^ of DPSCs were seeded in six wells plates with α-MEM, supplemented with 10% FCS, 100 μM l-ascorbic acid, 2 mM l-glutamine and penicillin (100U/mL)/streptomycin (100 mg/ml) and incubated from 24 h at 37°C with 5% CO_2_ for complete adhesion. Previous to the experiment cells were starved for 6hs in α-MEM without FCS. After starvation period, 10 μg/ml of LPS were added in α-MEM, supplemented with 10% FCS, 100 μM l-ascorbic acid, 2 mM l-glutamine and penicillin (100 U/mL)/streptomycin (100 mg/ml), for 24 hs.

Part of these cells was further irradiated (see PBM protocol following) with a laser device (Group 1); while the other part remained not irradiated for subsequent analysis (Group 2).

### Photobiomodulation Irradiation Protocol

DPSCs assigned for Group 1 were irradiated using the Laser Duo device (MM Optics, São Carlos, BR), containing 1 light-emitting diode, in the infrared wavelength (808 nm) that delivered a total energy of 6 J (100 mW × 60 s). 0.4 × 10^5^ cells per well were seeded in a 96 wells plate 24 hs before the experiment. All the three experimental groups were evaluated in triplicates. The plate cover and the culture medium were removed prior irradiation to avoid any interference related to light refraction. The laser probe was placed perpendicularly from bottom of the well with a 0.5 cm of distance for 60 s, in order to be sure that all cells received the radiation. After the treatment, the culture medium was replaced for 30 min before the genetic material be assessed. Device specifications: laser type, InGaAIP; wavelength, 808 nm; irradiation type, Infrared; laser beam output area, 0.3 cm^2^; Continuous; power output, 100 mW ± 20%; irradiance, 0.33 W/cm^2^; fluence, 6 J/cm^2^ (Table 1).

**TABLE 1:** mRNA expression of cell adhesion and extracellular matrix proteins in DPSCs after and before laser irradiation.

Gene	LPS	LPS + Laser	Gene Fold (Change Factor)
ACVR1	1.15	0.66	−1.74
AHSG	1.21	0.53	−2.28*
ALPL	2.85	6.15	2.16*
ANXA5	1.99	1.48	−1.34
BGLAP	0.61	0.26	−2.35*
BGN	1.31	1.13	−1.16
CDH11	1.33	1.30	−1.02
CD36	0.11	0.06	−1.83
COL10A1	2.89	5.84	2.02*
COL14A1	0.82	0.79	−1.04
COL15A1	1.21	1.27	1.04
COL1A1	1.12	0.83	−1.35
COL1A2	1.00	0.77	−1.29
COL2A1	1.21	0.53	−2.28*
COL3A1	1.02	0.99	−1.03
COL5A1	1.65	0.98	−1.68
COMP	1,21	0.53	−2,28*
FN1	1.04	0.83	−1.25
ICAM1	29.32	30.55	1.04
ITGA1	4.73	2.98	−1.59
ITGA2	3.20	1.95	−1.64
ITGA3	0.61	0.60	−1.02
ITGAM	1.96	0.53	−3.70*
ITGB1	2.00	1.48	−1.35
SPP1	0.95	0.56	−1.70
VCAM1	6.07	3.67	−1.65

### Proteinase-Activated Receptors Gene Expression Quantification

The tRNA from DPSCs was extracted using the TRIzol^®^ (Thermo Fischer Scientific, Waltham, United States) reagent. The complementary DNA (cDNA) was obtained from the tRNA by reverse-transcription using the ImProm-II Reverse transcriptase System kit (Promega, Madison, United States) according to the manufacturer’s protocol. The gene expression evaluation was performed by Real-time quantitative polymerase chain reaction (qRT-PCR) assays, using the SYBR Green PCR Master Mix® (Thermo Fisher Scientific, Waltham, United States). The reaction cycling parameters were adjusted to 50°C for 2 min and 95°C for 10 min, followed by 40 cycles at 95°C for 15 s and 60°C for 1 min in an Real Time PCR System ABI PRISM 7500 (Applied Biosystems, Foster City, United States). Relative quantification was carried out using the ΔCt method. This method results in ratios between the target genes and the housekeeping reference gene, in this case, the enzyme β-2-microglobulin (positive control) for DSPCs. This assay is based on gene fold-Regulation approach, that represents in fold-change (fc) results of the evaluated genes. Fold-change values greater than one indicates a positive- or an up-regulation in gene expression, while fold-change values less than one indicate a negative or down-regulation, of the evaluated gene. The *p*-values were calculated based on a Student’s t-test of the replicate 2^ (ΔCt) values for each gene in the experimental or control groups. All primers sequences used in PARs genes evaluation were manufactured by Exxtend (São Paulo, SP, Brazil). The melting curve analysis was used to determine the specificity of the reaction. All experiments were run in triplicates.

### Inflammation and Osteogeneses Genes Expression Array

Likewise, DPSCs from the 3 experimental groups were assessed for expression of eighty-five (85) genes related to inflammation and osteogenesis the RT^2^ Profiler ™ PCR array (Qiagen, Hilden, GER) was used. This device allows gene expression profiling technology by analyzing focused panels of genes using real-time PCR. Each experimental kit contains a set of the inflammatory/osteogenesis pathway-converged genes, and five housekeeping (positive controls) genes. The array also contains a panel of other reaction controls to monitor real-time PCR efficiency (PPC), genomic DNA contamination (GDC) as well as the first strand synthesis (RTC). All experiments were run in triplicates.

### Statistical Analysis

Raw data for each experiment (PARs, inflammation and osteogenesis gene expression) was transformed in a fractional number as function of the data from control group, using a single rule of 3, wherein the gene expression values of control groups for each experiment were considered = 1. Lack of normal distribution demanded the application of non-parametric Kruskal–Wallis tests, complemented by Mann–Whitney tests for pairwise comparison, at a 5% level of significance.

## Results

### Proteinase-Activated Receptor Expression

**FIGURE 1:**
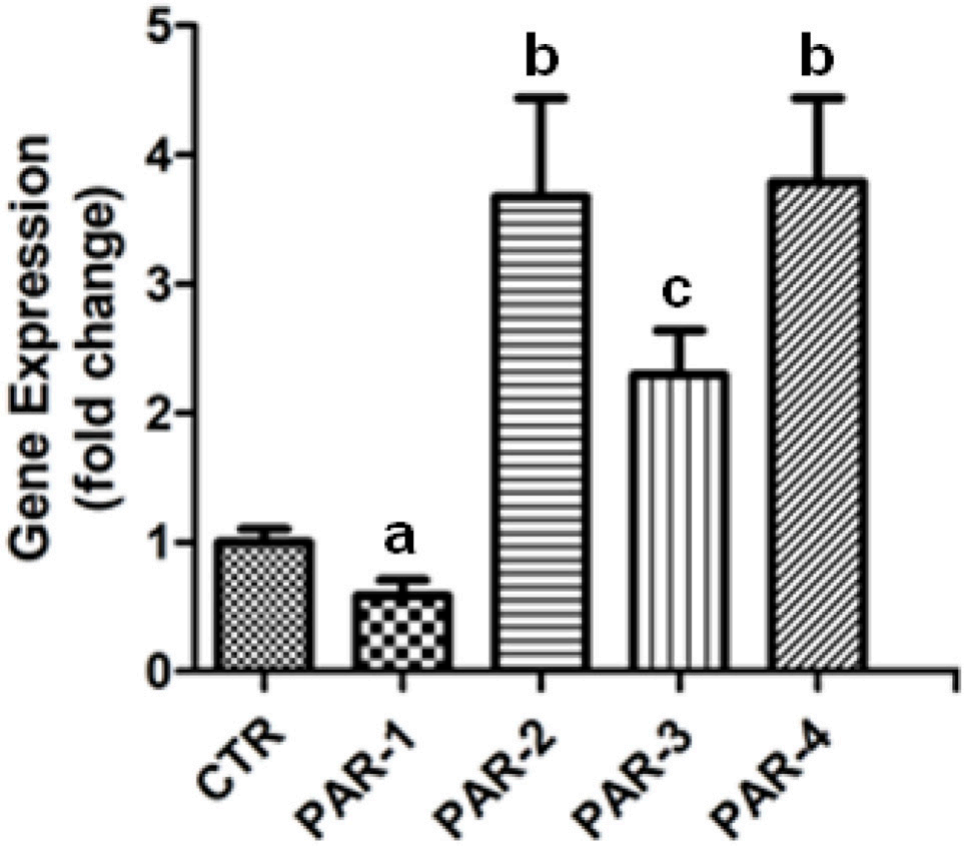
PBM modulate PARs genes expression in DPSCs. Real-time quantitative PCR (qRT-PCR) analysis of gene expression in LPS stimulated DPSCs irradiated with the infrared wavelength (808 nm) with total energy of 6 J (100mW × 60s). The bars represent the mean expression levels of the four PARs genes and standard error. The qRT-PCR analysis of gene expressions was normalized to the housekeeping gene enzyme β-2-microglobulin for DPSCs (control); the relative quantification of the expression levels (experimental/control) was determined based on the 2-ΔCt method. Experiments were performed in triplicate. Different letters indicate statistically significant differences (*p < 0.05*) in gene expression.

Four PARs have been identified so far (PAR-1–4) and evidence shows that they can exhibit both anti- and pro-inflammatory properties. Figure 1 shows that PBM treatment can modulate the gene expression of all PARs members. While PAR-1 showed a downregulation in the gene expression, the other PARs show a significant increase in the number of gene copies.

### Photobiomodulation Effect on Gene Expression of Human Dental Pulp Stem Cells After Lipopolysaccharide Inflammatory Stimulus

PBM therapy showed a wide range of effects at the tissue, cellular, and molecular levels. LPS-inflamed DPSCs demonstrated to be sensitive to PBM irradiation with several genes related to inflammation and osteogenesis being modulated either up or down after treatment. Among the genes analyzed, Table 1 depicts the DPSCs genes related to proteins involved in the extracellular matrix (ECM) organization that were modulated by LPS inducement and/or then regulated or inhibited by PBM irradiation.

### Photobiomodulation Effect on Expression of Proteolytic Enzymes Genes of Human Dental Pulp Stem Cells

Both *in vivo* and *in vitro* have used PBM because it is an important tool to positively stimulate bone. However, little is known about its association with anti-inflammatory and neoformative events taking place in human stem cells derived from a mineralized tissue. The data presented in Table 2 show the expression of proteolytic enzymes genes related to organic matrix remodeling in mineralized tissues that were upregulated in the inflammatory state and then, mainly downregulated after PBM therapy. From all enzymes evaluated only MMP-10 showed an increase in the expression (1.81-fold), while MMP-8 and MMP-9 gene expression were significantly reduced -3.03 and -2.68-fold, respectively.

**TABLE 2:** mRNA expression of proteolytic enzymes related to organic matrix remodeling in the DPSCs before and after laser irradiation.

Gene	LPS	LPS + Laser	Gene Fold (Change Factor)
CTSK	1,70	1.14	−1.49
MMP2	1.30	0.96	−1.35
MMP8	1.60	0.53	−3.02*
MMP9	1.42	0.53	−2.68*
MMP10	0.54	0.98	1.81
PHEX	0.26	0.18	−1.44
SERPINH1	1.30	0.99	−1.31

### Photobiomodulation Effects on Genes Expression of Growth and Differentiation Factors in Human Dental Pulp Stem Cells

Initially known as an active secreted molecule that can affect the growth of the cells, the growth factors family has been expanded his activities to include secreted molecules that can affect cellular differentiation and promote or inhibit mitosis. Table 3 show the expression profile of genes that can act on specific cell surface receptors that subsequently transmit their growth signals to other intracellular components, after inflammatory stimulus, and after PBM treatment. Our results clearly show that the growth factors were predominantly upregulated by LPS, while PBM treatment lowered the gene expression of proteins related to tissue mineralization, such as BMP-1 (-3.67-fold), BMP-4 (-3.73-fold), and BMP-7 (-2.28-fold). In the same sense, VEGF-B was significantly downregulated by laser treatment (-9.43-fold).

**TABLE 3:** mRNA expression of growth and differentiation factors in the DPSCs before and after laser irradiation.

Gene	LPS	LPS + Laser	Gene Fold (Change Factor)
BMP1	3.05	0.83	−3.67*
BMP2	2.85	1.82	−1.47
BMP3	1.21	1.94	1.60
BMP4	1.53	0.41	−3.73*
BMP5	0.52	0.57	1.90
BMP6	1.50	1.59	1.06
BMP7	1.21	0.53	−2.28*
CHRD	1.21	0.53	−2.28*
CSF1	1.36	1.28	−1.06
CSF2	0.13	0.16	1.23
CSF3	0.77	0.49	−1.57
EGF	1.21	1.53	1.26
FGF1	3.51	1.29	−2.72*
FGF2	2.63	2.09	−1.26
GDF10	1.21	0.53	−2.28*
IGF1	0.37	0.51	1.38
IGF2	1.21	0.53	−2.28*
IHH	1.21	0.53	−2.28*
NOG	2.80	2.79	−1.00
PDGFA	1.22	1.24	1.01
TGFB1	1.79	0.88	−2.03*
TGFB2	2.19	1.12	−1.96
TGFB3	0.47	0.52	1.11
TNF	1.21	0.53	−2.28*
VEGFA	1.52	1.16	−1.31
VEGFB	2.64	0.28	−9.43*

### Photobiomodulation-Modulating Effect on Gene Expression of Cell Surface Receptors

Once the expression of growth factors was evaluated, the gene expression for cell receptors related to these specific ligands was also evaluated. As expected, the expression of the cellular receptors followed the trend profiling found as far expression of their respective ligands. For most of evaluated cellular receptors, it was observed an increase in their gene expression after induction with LPS, while PBM treatment downregulated all evaluated genes. Interestingly, the most significantly inhibited receptors were those related to tissue mineralization processes (Table 4), including: Calcium Receptor (-2.28-fold), and Vitamin D Receptor (-37.66-fold).

**TABLE 4:** mRNA Expression of Cellular Receptors Related to Osteogenesis in the DPSCs After and Before Laser Irradiation.

Gene	LPS	LPS + Laser	Gene Fold (Change Factor)
ACVR1	1.15	0.66	−1.74
BMPR1A	2.02	1.40	−1.44
BMPR1B	1.11	0.55	−2.02*
BMPR2	1.81	1.40	−1.29
CALCR	1.21	0.53	−2.28*
EGFR	1.33	1.11	−1.20
FGFR1	1.24	0.95	−1.31
FGFR2	0.78	0.13	−6.00*
FLT1	0.19	0.55	2.89*
IGF1R	3.88	1.90	−2.04*
TGFBR1	1.36	0.92	−1.48
TGFBR2	1.31	0.81	−1.62
TNFSF11	0.58	0.37	−1.57
VDR	15.44	0.41	−37.66*

(ACVR1) Activin A receptor type I. (BMPR1A) Bone morphogenetic protein receptor, type IA. (BMPR1B) Bone morphogenetic protein receptor, type IB. (BMPR2) Bone morphogenetic protein receptor, type II. (CALCR) Calcitonin receptor. (EGFR) Epidermal growth factor receptor. (FGFR1) Fibroblast growth factor receptor 1. (FGFR2) Fibroblast growth factor receptor 2. (FLT1) Fms-related tyrosine kinase. (IGF1R) Insulin-like growth factor 1 receptor. (TGFBR1) Transforming growth factor, beta receptor 1. (TGFBR2) Transforming growth factor, beta receptor II. (TNFSF11) Tumor necrosis factor (ligand) superfamily, member 11. (VDR) Vitamin D (1.25- dihydroxyvitamin D3) receptor. The numbers represent the quantification of the gene expression variation in fold change, and the * represents genes that were modulated + or – 2.00-fold *(p<0.05)*.

### Photobiomodulation-Modulating Effect on Gene Expression of Transcription Factors

Transcription factors are proteins that regulates the transcription of genes. They have DNA-binding sequences that give them the ability to bind to specific of DNA domains called enhancer sequences. The evaluated members of this family were basically not modulated by LPS (Table 5). Except for the SOX-9 gene that showed a 3.68-fold increase in expression levels. Conversely, the PBM treatment downregulated the expression of all analyzed genes with the SP-7 (−3.28-fold), GLI-1 (−2.85-fold), and TWIST-1 (−2.35-fold) showing to be the most sensitive genes to laser irradiation.

**TABLE 5:** mRNA expression of transcription factors in the DPSCs before and after laser irradiation.

Gene	LPS	LPS + Laser	Gene Fold (Change Factor)
DLX5	1.21	0.53	−2.28*
GLI1	1.51	0.53	−2.85*
NFKB1	1.21	1.27	1.05
RUNX2	0.83	0.80	−1.04
SMAD1	1.51	0.66	−2.29*
SMAD2	1.68	1.09	−1.54
SMAD3	1.55	0.79	−1.96
SMAD4	2.80	1.36	−2.06*
SMAD5	1.99	1.76	−1.13
SOX9	3.68	3.20	−1.15
SP7	1.74	0.53	−3.28*
TWIST1	1.56	3.78	2.35*

## Discussion

In the regenerative dentistry field researchers face two major challenges, both related to the pulp tissue pathophysiology. The first is the tissue ability to turn back to a healthy metabolic state after a transitory inflammation. The second concerns to its intrinsic potential to produce mineralized tissue through its specialized cells, the odontoblasts. The appreciation of human dental pulp stem cells (DPSCs) as suitable for dental tissue engineering applications (Kaigler and Mooney, 2001; Young et al., 2005; Duailibi et al., 2008; Zhang et al., 2009) renders the overall ambition of tissue regeneration in dentistry more accomplishable. The PBM therapy or PBM treatment has been used in medicine and dentistry for its well demonstrated analgesic, anti-inflammatory, and biostimulation effects (Reddy, 2004; Silveira et al., 2007; Moosavi et al., 2016). In addition, the present study has shown, for the first time, that such modulatory physio-biological effects of PBM is accompanied by a modulation on the gene expression of inflammation and osteogenic biomarkers in DPSCs.

Proteinase-activated receptors (PARs) have a distinct mechanism of activation that involves limited proteolysis and unmasking of a receptor activating motif called tethered ligand (Huesa et al., 2016). PAR-1 and PAR-4 are canonically described as thrombin-activated receptors, while PAR-2 is activated by trypsin. Interestingly, PAR-3 was related to play role during embryonic development (Gieseler et al., 2013). Our results clearly showed that PBM therapy can increase the expression of PAR-2 (3.7-fold), PAR-3 (2.3-fold), and PAR-4 (3.8-fold) while decreasing the expression of PAR-1 (-0.6-fold). Interestingly, matrix metalloproteinases (MMPs), which genes have shown to be upregulated by PBM in the present study, have been described to activate PARs in a noncanonical way (Alvarez et al., 2017). Thus, taken in conjunction, these results strongly suggest that PARs can play a role in the anti-inflammatory process promoted by PBM therapy by not only increasing their expression, but also by increasing the MMPs expression levels.

Even under basal conditions DPSCs produce these molecular markers, including but not limited to CD-36, BMPs, NOG, Type II collagen, RUNX2, SOX-9 that are responsible for maintenance of pluripotency in early embryos and embryonic stem cells (Govindasamy et al., 2010; Karaoz et al., 2010; Martinez-Sarra et al., 2017), which indicate a promising primitiveness and multipotency of DPSCs for regenerative dentistry.

The osteogenic gene profile analysis was performed to provide better understanding on the underlying mechanisms for DPSCs differentiation and modulation of mineralization process upon the effects of an inflammatory model (LPS) and PBM (Tables 2–5). The treatment of DPSCs with LPS was capable to remarkably induce the gene expression of odontoblastic differentiation biomarkers, BMP-1, BMP-2, BMPR-1A, FGF-1, FGF-2, TGF-B2, IGF-1R, SMAD-4, SMAD-5, COL-10A1, ITG-A1, ITG-A2, ITG-B, ITG-A, and Alkaline phosphatase (ALP) in DPSCs. ALP is a widely accepted as an earlier marker for the differentiation of cells forming mineralized tissues. It has been shown that BMP-2 is required to induce the differentiation of DPSCs into odontoblast (Casagrande et al., 2010), and in mesenchymal stem cells, BMP-2 efficiently induced the expression of transcriptional factor Sox9 responsible for chondrogenic differentiation via BMP-2/Smad (Pan et al., 2008). Transforming growth factor-beta (TGF-β), also via SMAD pathways, plays a major role in tooth development and the reparative process by regulating cell proliferation, differentiation, and reparative dentinogenesis (Niwa et al., 2018). FGF-1 and TGF-β1 have a synergic effect to promote morphological and functional features of differentiated odontoblasts, whereas FGF-2 seems to modulate TGF-β1 action (Unda et al., 2000). TGF-β1 and TGF-β3 are predominantly expressed in odontoblasts, whereas TGF-β2 is high expressed in dental pulp (Niwa et al., 2018). IGF-I stimulate osteoblast differentiation in human mesenchymal stem cells (HMSCs), it stimulates the biosynthesis of 1α.25(OH)2D in synergy with 25OHD3. Osteoblast differentiation and skeletal homeostasis may be regulated by autocrine/paracrine actions of 25(OH)D (3) in HMSCs (Geng et al., 2011). Here, we demonstrate that LPS can increases both IGF-1R and VDR expression in DPSCs favoring odontoblast differentiation. Taken together, our data suggest that LPS treatment induced odontoblastic differentiation of human DPSCs.

RANKL is a tumor necrosis factor (TNF)-like factor produced by mesenchymal cells, osteoblast derivatives, and T cells that is essential for osteoclastogenesis. In osteoblasts, RANKL expression is regulated by two major calcemic hormones, 1.25-dihydroxyvitamin D (3) [1.25(OH) (2)D (3)] and parathyroid hormone (PTH), as well as by several inflammatory/osteoclastogenic cytokines (Kim et al., 2006).

It is important to mention that 1.25(OH)2 vitamin D stimulates osteoblast maturation, increasing expression of the mature osteoblast marker osteocalcin (BGLAP) in osteoblasts, and 1.25(OH)2 vitamin D also stimulates the expression of the osteoclast differentiation factor RANKL (Pereira et al., 2019). The biological actions of 1.25-(OH)2D3 are mediated by the vitamin D receptor (VDR), a protein that binds to target genes and alters their expression. 1.25-(OH)2D3 is also able of inducing transcription of the VDR gene itself (Zella et al., 2006). VDR signaling in osteoprogenitors cells increases RANKL expression and stimulates osteoclastogenesis (Kim et al., 2006).

In this sense, PBM treatment significantly suppressed the mRNA expression of TNF and RANKL (TNFSF11) triggered by LPS in DSPC. Interesting, PBM treatment greatly decreased (37.66-fold) the expression Vitamin D Receptor (VDR) triggered by LPS in the cell model. PBM treatment can inhibit the transcriptional activity of NF-κB in human periodontal ligament cells (Lee et al., 2018), which is a crucial transcription factor involved in the regulation of the inflammatory process triggered by LPS in dental pulp stem cells (Baldwin, 2001). PBM decreased cell death and attenuated the NLRP3 inflammasome in the ischemic brain. In mice experimental model with ischemic stroke, PBM therapy showed suppressed TLR-2 levels, MAPK signaling and NF-kB activation. The suppression of NF-kB activation induced by PBM is related to the major anti-inflammatory activity of Laser (Lee et al., 2017).

Our data suggest that the decrease in the VDR expression promoted by PBM treatment can lead to inhibition of 1.25(OH)_2_D_3_/VTR signaling, and downregulating RANKL expression in DPSCs (Kim et al., 2006). PBM therapy also decreased the expression of proteolytic enzymes cathepsin K, MMP-8 and MMP-9 after LPS assay. Overall, PBM blocked the odontoblastic differentiation of human dental pulp stem cells subjected to LPS assay that were dependent on BMP, FGF, IGF and TGFB signaling, decreasing the expression of the transcriptional factor DLX-5 and SP-7 as expected. SP-7 and Dlx5, in turn, was shown to drive the differentiation of mesenchymal precursor cells into osteoblasts (Nakashima et al., 2002). Conversely, it is important to mention that PBM did not block the mRNA expression of ALP, Twist homolog 1 (TWIST-1) and Runt-related transcription factor 2 (RUNX-2) in DPSCs, even in the presence of LPS. TWIST-1 protein regulates several genes that are known to be key players in bone formation, including the FGF-R2 and RUNX-2 genes. In conjunction, our data strongly suggest that PBM decreased inflammatory mineral matrix resorption in DPSCs by decreasing the activation of RANKL expression via inhibition of 1.25(OH)_2_D_3_/VTR signaling. Moreover, PBM treatment preserved the odontogenic potential of DPSCs by increasing the expression of TWIST-1/RUNEX-2/ALP signaling axis.

In spite of the study limitations, were possible to conclude that biomodulation of DPSCs by irradiation with laser device at the infrared wavelength (808 nm) showed not only to mediate the gene expression related to habitual anti-inflammatory molecular canons, but also participate in the regulation of genes that can express signaling molecules and factors associated with other non-classic inflammation molecules (i.e. PARs, cell receptors and transcription factors) as well as with molecules of bone tissue protection from resorption, even if it has not clearly shown yet to influence the expression of genes that stimulate bone neoformation. Further studies still need to be performed to better elucidate the role of PBM therapy on the DPSCs and bone. However, this study certainly brings new data to tissue engineering related to the regenerative pulp therapy field.

## Data Availability

The datasets presented in this study can be found in online repositories. The data can be accessed from the Gene Expression Omnibus (GEO) database, using accession number GSE193448.
